# Non-disclosure of drug injection practices as a barrier to HCV testing: results from the PrebupIV community-based research study

**DOI:** 10.1186/s12954-023-00841-7

**Published:** 2023-07-29

**Authors:** Ilhame Anwar, Cécile Donadille, Camelia Protopopescu, David Michels, Joris Herin, Adélaïde Pladys, Danièle Bader, Patrizia Carrieri, Perrine Roux

**Affiliations:** 1 Aix Marseille Univ, Inserm, IRD, SESSTIM, Sciences Economiques and Sociales de la Santé and Traitement de l’Information Médicale, ISSPAM, Marseille, France; 2 AIDES, Pantin, France; 3 Laboratoire de Recherche Communautaire, Coalition PLUS, Pantin, France; 4 Bus 31/32, Marseille, France; 5 Coordination Nationale des Réseaux de Microstructures (CNRMS), Strasbourg, France

**Keywords:** HCV testing, PWID, Testing, Disclosure, Stigma

## Abstract

**Background:**

Hepatitis C virus (HCV) infection prevalence is particularly high in people who inject drugs (PWID), a population that faces many barriers to HCV testing and care. A better understanding of the determinants of access to HCV testing is needed to improve their engagement in the HCV care cascade. We used data from a cross-sectional survey of people who inject drugs, mainly opioids, to identify factors associated with recent HCV testing.

**Methods:**

Self-reported data on HCV antibody testing were analyzed for 550 of the 557 PWID enrolled in PrebupIV, a French cross-sectional community-based survey which assessed PWID acceptability of injectable buprenorphine as a treatment. Factors associated with recent (i.e., in the previous six months) HCV antibody testing were identified performing multivariable logistic regression.

**Results:**

Among the study sample, 79% were men and 31% reported recent HCV antibody testing. Multivariable analysis found that PWID who did not disclose their injection practices to anyone (aOR [95% CI] 0.31 [0.12,0.82], *p* = 0.018), older PWID (aOR [95% CI] 0.97 [0.95,1.00], *p* = 0.030) and employed respondents (aOR [95% CI] 0.58 [0.37,0.92], *p* = 0.019) were all less likely to report recent HCV testing. No association was found between opioid agonist therapy and HCV testing.

**Conclusions:**

Our findings suggest that non-disclosure of injection practices, employment and age were all barriers to HCV antibody testing. Preventing stigma around injection practices, developing the HCV testing offer in primary care and addiction care services, and training healthcare providers in HCV care management could improve HCV testing and therefore, the HCV care cascade in PWID.

## Background

In people who inject drugs (PWID), hepatitis C virus (HCV) transmission mainly occurs through the sharing of contaminated injection materials [1]. In Western Europe, estimated HCV seroprevalence in PWID was 53.2% in 2017, accounting for approximately 537,000 individuals [2]. HCV prevalence in PWID in France ranges from 48 to 64% [3, 4], depending on the sample; this is considerably higher than the estimated 1% in the French general population [5]. Micro-elimination is a nested strategy of the World Health Organization (WHO) hepatitis C elimination plan [6] which aims to eliminate HCV epidemic by 2030 (90% and 65% reduction in incidence and mortality, respectively). France has planned to reach this goal by 2025 [7].

Despite the high prevalence of HCV in PWID in France, testing is insufficient. Consequently, a large proportion of PWID with the disease are undiagnosed [8–10]. A recent French study conducted in harm reduction services suggested that 8% of PWID who had injected at least once during their lifetime had never been tested for HCV. That study also suggested that only half of HCV antibody-negative (52%) people who use drugs [11] had been tested during the previous six months as per official recommendations in France [12]. Testing is a key stage in the HCV care cascade; increasing testing in terms of the number of people tested and the frequency can result in prompt HCV cure, a lower risk of transmission to other PWID, and a lower HCV-related morbidity burden [13].

Although direct-acting antivirals (DAAs) for HCV have led to considerably greater access to care for HCV-infected individuals, a substantial proportion of PWID do not yet benefit from this recent treatment. Furthermore, PWID face many barriers to HCV testing and care access at the individual, provider and health system levels. At the individual level, barriers include poor social conditions (unstable housing, lack of health insurance) and limited knowledge about HCV infection [14–16]. At the provider level, stigma and discrimination around drug injection play a large role in PWID underuse of available healthcare services [17–19]—including HCV testing [20]—and poor engagement in care [21]. Barriers at the health system level include limited geographical and financial accessibility to testing and criminalization of drug use [14, 15, 19, 22, 23]. For example, although integrated care could help to optimize the care cascade for PWID [12], onsite RNA testing or treatment are not systematically available in harm reduction or addiction care services in France [24]. In the context of simplifying HCV management, non-specialist primary care provider involvement [25], the HCV testing offer, and treatment uptake in prison settings [26] should all be reinforced. While certain health system barriers for PWID have been successfully tackled in recent years [12], many individual and provider-level barriers persist. These need to be explored in greater detail in order to better identify strategies to engage PWID in the HCV care cascade.

In this context, we used the PrebupIV study to identify factors associated with recent HCV antibody testing (i.e., during the previous six months) in PWID, mainly opioids, living in France.

## Methods

### Study design

PrebupIV is a cross-sectional community-based survey conducted between May and August 2015 in France in collaboration with the association AIDES and with the support of other associations (Psychoactif, Fédération Addiction, ASUD, Médecins du Monde). It aimed to assess PWID acceptability of intravenous buprenorphine as a treatment. The survey is described in detail elsewhere [27].

Eligibility criteria were as follows: 18 years of age or older, French speaking and having injected opioids in the previous week. The survey questionnaire was administered face to face by field workers in harm reduction services or was self-administered online using a web link available on Psychoactif.org. The contents of the questionnaire did not differ between the questionnaire types (i.e., face-to-face *versus* online). The questionnaire collected self-reported data about sociodemographic characteristics, behavioral and health data, drug use practices, and access to HCV testing. A total of 557 PWID completed the questionnaire. No reimbursement was provided for participation. The survey was authorized by the national French Data Protection Authority [*Commission Nationale de l'Informatique et des Libertés* (CNIL)] (approval number 1812588v0–05/12/2014). The protocol was designed in accordance with the 1964 Helsinki Declaration, and all participants provided informed consent prior to their inclusion.

### Study sample

**Fig. 1:**
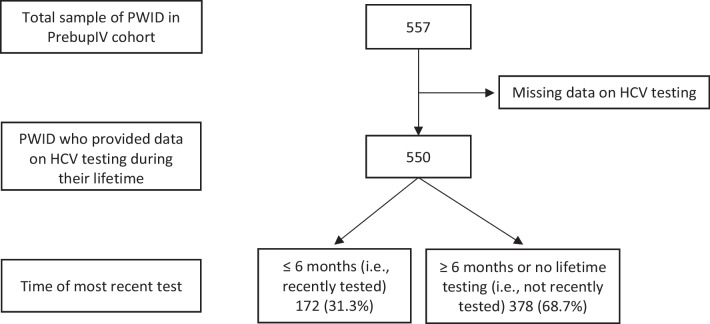
Flowchart for study sample

For the present study, we selected 550 PWID among PrebupIV’s 557 participants. People without data on history of HCV testing (*n* = 7) (Fig. 1) were excluded.

### Variables

The main outcome ‘recent HCV testing’ was created as a dichotomous variable by identifying participants who reported testing for HCV in the previous six months, and those tested either more than six months previously or never tested. People who answered “Do not know” were classified in the latter group. This variable was created in accordance with current French recommendations on HCV testing frequency for at-risk populations [12]. At the time of the study, only HCV antibody testing was available in harm reduction services; RNA testing was not available.

Independent variables were sociodemographic data (age, gender, unstable housing, employment), behavioral and health data [experience of recent incarceration, taking opioid agonist therapy (OAT)] and drug use practices (most frequently injected opioid, other injected substances, time since first injection of opioids, drug injection during the previous month, sharing of injecting equipment).

The variable ‘unstable housing’ included people living in a squat, or a caravan and those who reported being homeless (‘yes’ vs. ‘no’). The variable ‘recent incarceration’ included people who were incarcerated in the previous two years. The variable ‘currently on OAT’ reflected people who declared taking a prescribed OAT (buprenorphine, methadone, morphine sulfate) during the previous month; OAT was always prescribed in oral form. The variable ‘most frequently injected opioid’ was created considering the number of days of injection per month. The dichotomous variable ‘disclosure of injection practices’ comprised two categories: not having disclosed to anyone and having disclosed to someone, irrespective of whether this was a healthcare provider (e.g., addiction specialist, other specialist physician, nurse, general practitioner, pharmacist, harm reduction service worker) or other person (e.g., family, friend, internet forum).

### Statistical analyses

We described and compared participants recently tested with those who were not, using a Chi-square and Wilcoxon test for categorical and continuous data, respectively.

To identify factors associated with recent HCV testing, we first performed univariable logistic regressions to identify eligible variables for the multivariable model at a threshold *p* value < 0.20. We then performed multivariable logistic regression using a backward stepwise procedure. Only variables with a *p* value < 0.05 were retained in the final model. The latter was adjusted for the type of questionnaire to take into account differences between people recruited in harm reduction services and those recruited online (Psychoactif.org). All statistical analyses were performed with Stata SE 14.2 software (StataCorp. 2015. Stata Statistical Software: Release 14. College Station, TX: StataCorp LP).

## Results

### Descriptive analysis of the study sample

Table 1 presents the baseline characteristics of the 550 PWID in PrebupIV selected for the analyses.

**Table 1:** Descriptive analysis of study sample (*n* = 550)

	Not recently tested *n* = 378 (68.7%)	Recently tested *n* = 172 (31.3%)	*p* Value	Total *n* = 550
*Questionnaire*			0.005	
Online	121 (32.0)	35 (20.3)		156 (28.4)
Face-to-face	257 (68.0)	137 (79.7)		394 (71.6)
*Age (years)*			0.135	
Median [IQR]	34 [29–42]	33 [27–40]		34 [28–41]
*Gender*			0.289	
Men	296 (79.8)	143 (83.6)		439 (81.0)
Women	75 (20.2)	28 (16.4)		103 (19.0)
*Employment*			0.002	
Unemployed	244 (66.8)	136 (80.0)		380 (71.0)
Employed	121 (33.2)	34 (20.0)		155 (29.0)
*Unstable housing*			0.026	
No	299 (79.9)	122 (71.3)		421 (77.2)
Yes	75 (20.1)	49 (28.7)		124 (22.8)
*Recent incarceration*			0.018	
No	327 (89.1)	138 (81.7)		465 (86.8)
Yes	40 (10.9)	31 (18.3)		71 (13.2)
*Currently on OAT*			0.845	
No	109 (28.8)	51 (29.7)		160 (29.1)
Yes	269 (71.2)	121 (70.3)		390 (70.9)
*Opioid injected most frequently*			0.372	
Buprenorphine	173 (45.8)	81 (47.1)		254 (46.2)
Heroin	68 (18.0)	26 (15.1)		94 (17.1)
Morphine	58 (15.3)	26 (15.1)		84 (15.3)
Methadone	16 (4.2)	14 (8.1)		30 (5.5)
Other ^a^	63 (16.7)	25 (14.5)		88 (16.0)
*Other non-opioid substances injected*			0.111	
No	177 (46.8)	68 (39.5)		245 (44.5)
Yes	201 (53.2)	104 (60.5)		305 (55.5)
*Time since first opioid injection (years)*			0.1021	
Median [IQR]	7 [3–12]	6 [3–10]		7 [3–11]
*Drug injection frequency during the previous month (days)*			0.7861	
Median [IQR]	30 [20–30]	30 [15–30]		30 [20–30]
*Sharing injection equipment*			0.601	
No	302 (82.3)	143 (84.1)		445 (82.9)
Yes	65 (17.7)	27 (15.9)		92 (17.1)
*Disclosure of injection practices*^b^			0.001	
Yes	320 (88.2)	163 (97.0)		483 (91.0)
No	43 (11.8)	5 (3.0)		48 (9.0)

*HCV* hepatitis C virus, *IQR* interquartile range, *OAT* opioid agonist therapy, *p value* Chi-2 or Wilcoxon test

^a^Oxycodone, codeine, others and missing

^b^Addiction specialist, other specialist physician, nurse, general practitioner, pharmacist, harm reduction services worker, family, friends, internet forum

One third (31.3%) reported recent (i.e., in the previous six months) HCV testing, while 68.7% did not (i.e., tested more than six months previously or never tested).

Eighty (81.3%) percent were men and 18.7% women. Median age was 34 years [interquartile range (IQR): 28–41]. Twenty-two percent had unstable housing and 69% were unemployed. Thirteen percent reported recent incarceration (i.e., in the previous two years).

Buprenorphine was the opioid injected most frequently (46.2%), followed by heroin (17.1%) and morphine (15.3%). Over half (55.5%) the study sample reported injecting substances other than opioids (e.g., cocaine, amphetamines). Median time since first injection of opioids was seven years (IQR: 3–11). Seventy percent were currently on OAT and 17.1% reported sharing injection equipment. Nine percent had never disclosed their injection practices to anyone.

PWID recently tested for HCV were more likely to have answered the face-to-face questionnaire, to be unemployed, to have unstable housing, to be recently incarcerated, and to have talked with someone about their injection practices.

### Factors associated with HCV testing in the study sample

Table 2 presents the results of the univariable and multivariable regression analyses.

**Table 2:** Factors associated with recent HCV testing (*n* = 550); univariable and multivariable logistic regression analyses

	Univariable logistic regression			Multivariable logistic regression		
	OR	CI	*p*	aOR	CI	*p*
*Questionnaire*						
Online	1.00			1.00		
Face-to-face	1.84	[1.20,2.83]	0.005	1.65	[1.03,2.63]	0.037
*Age*	0.98	[0.96,1.00]	0.113	0.97	[0.95,1.00]	0.030
*Gender*						
Men	1.00					
Women	0.77	[0.48,1.25]	0.290			
*Employment*						
Unemployed	1.00			1.00		
Employed	0.50	[0.33,0.78]	0.002	0.58	[0.37,0.92]	0.019
*Unstable housing*						
No	1.00					
Yes	1.60	[1.06,2.43]	0.027			
*Recent incarceration*						
No	1.00					
Yes	1.84	[1.10,3.06]	0.019			
*Currently on OAT*						
No	1.00					
Yes	0.96	[0.65,1.43]	0.845			
*Opioid injected most frequently*						
Buprenorphine	1.00					
Heroin	0.82	[0.48,1.38]	0.448			
Morphine	0.96	[0.56,1.63]	0.873			
Methadone	1.87	[0.87,4.01]	0.109			
Other^a^	0.85	[0.50,1.44]	0.543			
*Other non-opioid substances injected*						
No	1.00					
Yes	1.35	[0.93,1.94]	0.111			
*Time since first opioid injection* (years)	0.97	[0.95,1.00]	0.090			
*Drug injection frequency during the previous month* (days)	1.00	[0.98,1.02]	0.764			
*Sharing injection equipment*						
No	1.00					
Yes	0.88	[0.54,1.43]	0.601			
*Disclosure of injection practices*^b^						
Yes	1.00			1.00		
No	0.23	[0.09,0.59]	0.002	0.31	[0.12,0.82]	0.018

*CI* confidence interval, *HCV* hepatitis C virus, *OR* odds ratio, *OAT* opioid agonist therapy

^a^Oxycodone, codeine, others and missing

^b^Addiction specialist, other specialist physician, nurse, general practitioner, pharmacist, harm reduction services worker, family, friends, internet forum

Univariable analyses showed that participants who answered the face-to-face questionnaire were more likely to have been recently tested for HCV (odds ratio (OR) [95% confidence interval (95% CI)] 1.84 [1.20,2.83], *p* = 0.005). They also highlighted a significant association between recent HCV testing and some sociodemographic characteristics. Employed PWID were less likely to have been recently tested for HCV than those who were unemployed (OR [95% CI] 0.50 [0.33,0.78], *p* = 0.002). Unstable housing (OR [95% CI] 1.60 [1.06,2.43], *p* = 0.027) and recent incarceration (OR [95% CI] 1.84 [1.10,3.06], *p* = 0.019) were associated with a greater likelihood of recent HCV testing. PWID who had not disclosed their injection practices to anyone were less likely to have been recently tested (OR [95% CI] 0.23 [0.09,0.59], *p* = 0.002) than those who had disclosed them. No association was found between being on OAT and recent testing for HCV.

In the multivariable analysis, after adjusting for the type of questionnaire (face-to-face *versus* online), older (aOR [95% CI] 0.97 [0.95,1.00], *p* = 0.030) and employed (aOR [95% CI] 0.58 [0.37,0.92], *p* = 0.019) PWID were less likely to have been recently tested for HCV. PWID who had not disclosed their injection practices to anyone were also less likely to have been recently tested (aOR [95% CI] 0.31 [0.12,0.82], *p* = 0.018) than those who had disclosed them.

## Discussion

The main finding from our analyses is that the non-disclosure of injection practices—whether to a healthcare professional or other person—was associated with less HCV testing in our study sample of PWID. This suggests that a taboo surrounding drug injection exists and that this taboo limits health promotion and PWID empowerment. The literature highlights that previous negative experiences with healthcare providers and the fear of being stigmatized or being treated poorly by them are huge barriers to testing and treatment for PWID [14, 15, 19]. Current and former PWID adopt strategies to avoid stigma and discrimination, including delaying healthcare as much as possible and not disclosing their drug use [28, 29]. In terms of HCV, this can lead to delayed testing and diagnosis as well as unwillingness to seek healthcare once diagnosed [20, 30]. A non-judgmental trustful healthcare provider-PWID relationship can facilitate HCV testing uptake [15, 30]. However, some healthcare providers feel that they do not have enough training to adequately consult PWID [31] or to manage HCV care for them (i.e., testing, diagnosis, liver disease assessment, treatment) [32]. Improved training of healthcare providers could change their representations and stereotypes of PWID. This could reduce stigma and discrimination and consequently improve the provider-patient relationship.

Employed PWID and older PWID were less likely to have been recently tested for HCV in our study. This suggests that employed PWID may not attend harm reduction services (where HCV testing is part of routine practice), a hypothesis supported by data from another study indicating that French harm reduction services mostly receive individuals living in social precarity [11]. Employed PWID probably attended primary care services more frequently, a setting where HCV testing is not routinely proposed. With regard to older PWID, our findings contradict previous French data which suggested that PWID under 25 years old was less frequently tested for HCV in harm reduction services than older persons [11]. One explanation for this contradiction may be that there was a lower prevalence of risk practices in older PWID in our study [33]. Another is that older PWID may have a more stable socioeconomic situation, which could translate into less use of harm reduction services in favor of primary care.

Moreover, participants in our study sample who answered the face-to-face questionnaire (i.e., recruited in harm reduction services) were more likely to have been recently tested for HCV. This is not surprising given that access to HCV testing is routine practice in these services (unlike in primary care).

Indeed, since 2016, the ‘targeted testing strategy’ has been recommended in France for people at risk of HCV contamination. Point-of-care (POC) testing strategies are also encouraged to facilitate access to HCV testing for marginalized PWID who do not attend healthcare facilities (primary care, hospitals, etc.) and for PWID who attend harm reduction services or primary care services but are at high risk of HCV infection [34].

In this context, innovative testing practices should be considered, such as point-of-care (POC) HCV RNA testing, in order to improve access to HCV testing for PWID who attend harm reduction centers and POC HCV antibody testing for those attending primary care services. A recent meta-analysis found that the use of onsite POC RNA viral load had a positive impact on reduced turnaround times between HCV antibody testing and treatment initiation, and on testing and treatment uptake for PWID, especially when it was proposed at the same visit and on the same day [35]. In France, a recent study demonstrated the feasibility and acceptability of a ‘test and treat’ strategy based on dedicated screening days, proposing both HCV antibody and RNA testing in addiction care centers [36]. More generally, combining ‘test and treat’ strategies, linkage to care and early treatment initiation, would be a cost-effective option for reducing HCV incidence and improving PWID life expectancy in the French context [37]. POC antibody and RNA testing should be proposed in primary care settings, particularly for people at risk of HCV infection, as primary care providers can initiate HCV treatment in the context of simplified HCV management.

Finally, unlike other studies, our results did not find any association between OAT receipt and recent testing for HCV [38–40]. A recent French study found that access to HCV treatment for people with opioid use disorders was proportional to the number of hepatologists and gastroenterologists in an area [41]. This would suggest a lack of involvement of primary care providers or addiction physicians in the HCV cascade of care. Previous studies in contexts outside France where primary care physicians are more involved in HCV care, suggested that primary care represented an excellent opportunity for HCV testing for PWID [15, 39] and that OAT receipt was associated with a greater likelihood of having been tested [40, 42]. These results highlight the importance of proposing testing in settings where OAT is prescribed. The training of primary care providers should be fostered and specialized centers for addiction care promoted, especially given that DAAs can be initiated in both these medical settings, thanks to the recent (2019) simplification of HCV management in France which permits primary care providers to prescribe and manage DAA-based HCV treatment [43]. Reinforcing cooperation between specialists and primary care providers could also be a lever to improve the HCV care cascade for PWID.

The PrebupIV study highlighted very good acceptability by PWID of an injectable treatment for opioid use disorder [27]. Developing such a treatment in France could empower PWID to talk more about their injection practices. In turn, this could help healthcare providers to identify at-risk practices and consequently offer HCV testing.

A primary care network called ‘microstructures’ has been in place for several years in France. It provides tailored primary care to people who use drugs characterized by psychosocial vulnerabilities [44, 45]. These medical microstructures, which are less stigmatizing than harm reduction services, may be more adapted for PWID who do not attend harm reduction services. Developing this offer and making it more visible for PWID could improve HCV testing uptake and therefore the HCV cascade of care. Further studies are needed to confirm these possibilities.

The present study has limitations. First, responses to the questionnaires were self-reported leading to possible social desirability bias. However, the validity and reliability of self-reports in terms of drug use among PWID were previously demonstrated in a literature review. It analyzed studies which assessed these two dimensions using test–retest methods or comparisons of urinalysis results, respectively [46]. Second, the study’s cross-sectional design and lack of randomization means that the study sample may not be representative of all French PWID. However, people who inject opioids represent the majority of PWID [11]. Third, women were under-represented in our sample (20%). Finally, the study was conducted in 2015 before universal access to DAAs; the French context may have changed since then. Having said that, access to HCV testing is still very much a multi-dimensional issue today for PWID [47], especially healthcare providers’ stigmatization of this population.

## Conclusions

In conclusion, in our study sample of PWID, non-disclosure of injection practices constituted a barrier to accessing HCV testing; this barrier may be influenced by healthcare providers’ behaviors. Employment and age were also individual barriers to HCV testing and should be taken into consideration when investigating access to HCV testing in this population. No association was found between being on OAT and HCV testing. Removing the stigma surrounding injection practices, developing a HCV testing offers in routine primary care and addiction care services, and training healthcare providers in HCV care management are all actions which could enhance HCV testing in PWID and so improve their HCV care cascade.

## Data Availability

The dataset used during the current study is available from the corresponding author upon reasonable request. IA had full access to all the data in the study and takes responsibility for the integrity of the data and the accuracy of the data analysis.

## Abbreviations

aOR: Adjusted odds ratio
DAAs: Direct-acting antiviral agents
HCV: Hepatitis C virus
IQR: Interquartile range
OR: Odds ratio
OAT: Opioid agonist therapy
POC testing: Point-of-care testing
PWID: People who inject drugs
RNA: Ribonucleic acid
