# Correction: Non‑disclosure of drug injection practices as a barrier to HCV testing: results from the PrebupIV community‑based research study

**DOI:** 10.1186/s12954-023-00848-0

**Published:** 2023-08-10

**Authors:** Ilhame Anwar, Cécile Donadille, Camelia Protopopescu, David Michels, Joris Herin, Adélaïde Pladys, Danièle Bader, Patrizia Carrieri, Perrine Roux

**Affiliations:** 1 Inserm, IRD, Aix Marseille Univ, SESSTIM, Sciences Economiques and Sociales de La Santé and Traitement de L’Information Médicale, ISSPAM, Marseille, France; 2 AIDES, Pantin, France; 3 Laboratoire de Recherche Communautaire, Coalition PLUS, Pantin, France; 4 Bus 31/32, Marseille, France; 5 Coordination Nationale Des Réseaux de Microstructures (CNRMS), Strasbourg, France

**Correction : Harm Reduction Journal (2023) 20:98** 10.1186/s12954-023-00841-7

Following publication of the original article [1], in this article first name and given name of author “Ilhame Anwar” has been interchanged, now it has been updated.

The original article has been corrected.
